# MMP-8 Deficiency Increases TLR/RAGE Ligands S100A8 and S100A9 and Exacerbates Lung Inflammation during Endotoxemia

**DOI:** 10.1371/journal.pone.0039940

**Published:** 2012-06-29

**Authors:** Adrián González-López, Alina Aguirre, Inés López-Alonso, Laura Amado, Aurora Astudillo, María Soledad Fernández-García, María F. Suárez, Estefanía Batalla-Solís, Enrique Colado, Guillermo M. Albaiceta

**Affiliations:** 1 Departamento de Biología Funcional, Instituto Universitario de Oncología del Principado de Asturias, Universidad de Oviedo, Oviedo, Spain; 2 Servicio de Medicina Intensiva, Hospital Universitario Central de Asturias, Oviedo, Spain; 3 Servicio de Anatomía Patológica, Hospital Universitario Central de Asturias, Oviedo, Spain; 4 Departamento de Bioquímica y Biología Molecular, Instituto Universitario de Oncología del Principado de Asturias, Universidad de Oviedo, Oviedo, Spain; 5 Servicio de Hematología y Hemoterapia, Hospital Universitario Central de Asturias, Oviedo, Spain; 6 CIBER-Enfermedades Respiratorias, Instituto de Salud Carlos III, Madrid, Spain; University of Pittsburgh, United States of America

## Abstract

Matrix metalloproteinase-8, released mainly from neutrophils, is a critical regulator of the inflammatory response by its ability to cleave multiple mediators. Herein, we report the results of a model of endotoxemia after intraperitoneal LPS injection in mice lacking MMP-8 and their wildtype counterparts. Control, saline-treated animals showed no differences between genotypes. However, there was an increased lung inflammatory response, with a prominent neutrophilic infiltration in mutant animals after LPS treatment. Using a proteomic approach, we identify alarmins S100A8 and S100A9 as two of the main differences between genotypes. Mice lacking MMP-8 showed a significant increase in these two molecules in lung homogenates, but not in spleen and serum. Mice lacking MMP-8 also showed an increase in MIP-1α levels and a marked activation of the non-canonical NF-κB pathway, with no differences in CXC-chemokines such as MIP-2 or LIX. These results show that MMP-8 can modulate the levels of S100A8 and S100A9 and its absence promotes the lung inflammatory response during endotoxemia.

## Introduction

The inflammatory response consists not only in local inflammation. In severe cases, this response spreads from the site of onset and evolves into a systemic injury [1]. In this setting, the lungs are amongst the most commonly involved organs. Neutrophils are recruited from the circulation [2], and a full-blown immune response takes place in both the interstitium and the alveolar spaces. This syndrome has been termed acute lung injury and may result in a severe lung dysfunction by altering gas exchange and respiratory mechanics. In patients with the systemic inflammatory response syndrome, lung injury is related to a high mortality rate [3].

The regulatory mechanisms responsible for the switch from a local to a systemic response are only partially known. There is increasing evidence that matrix metalloproteinases, a family of enzymes with a great variety of substrates, may modulate the inflammatory response by cleaving immune mediators and regulating cell migration [4]. Matrix metalloproteinase-8, also known as collagenase-2 or neutrophil collagenase, plays different roles in the regulation of the inflammatory response [5]. Mice lacking this enzyme show a delayed onset and also a slow clearance of the local inflammatory infiltrates [6], [7]. Several immune mediators, such as MIP-1α [8], IL-10 [9] or LIX [10], have been involved in this characteristic pattern and shown to be substrates of MMP-8.

We hypothesized that MMP-8 plays also a role in the lung response to endotoxemia. To test this hypothesis we used an experimental model of inflammation by intraperitoneal injection of lipopolysaccharide in wildtype and MMP-8 deficient mice. After documentation of increased neutrophil recruitment in the lungs from knockout mice compared to wildtype animals, we used a proteomic approach to identify the molecules involved in the observed differences in mice from both genotypes. These studies revealed the alarmins S100A8 and S100A9, which are significantly increased in mice lacking MMP-8, as two of the candidates to be responsible for the increased inflammatory response in mutant mice.

## Methods

### Animals

Mice deficient in MMP-8 were generated as previously described [6] and backcrossed to obtain a pure C57BL6 background. Normal mice with the same C57BL6 background were used as wildtype counterparts. Seventy-two animals were used in the study. Genotypes were confirmed by PCR in all animals. Animals were kept in SPF conditions, with 12∶12 hours light/dark cycles and free access to water and food. All the experimental protocols were reviewed and approved by the University of Oviedo Animal Research Ethics committee.

### Experimental Model

A dose of 5 mg/Kg of lipopolysaccharide (serotype O55:B5, Sigma-Aldrich) was intraperitoneally injected to wildtype and knockout mice. This dose induces lung inflammation with a peak 24 hours after injection [11]. Control animals from both genotypes were injected only with vehicle (sterile saline). After 24 hours, mice were anesthetized with a mixture of ketamine and xylazine, a laparotomy was performed and the animals were sacrificed by exsanguination. The lungs and the spleen were then removed. The right lung and the spleen were frozen at −80°C for further analysis. The left lung was fixated by intratracheal administration of 4% formaldehyde and immersed in the same fixative. In additional animals, a blood sample was obtained by cardiac puncture.

### Histological Study

Paraffin embedded sections were stained using a standard hematoxylin-eosin technique. Three sections per mouse were evaluated by two independent pathologists (AA, MSFG), blinded to the experimental conditions. Each section was scored from 0 to 3 based on the septal thickening (grade 1), areas of alveolar flooding (grade 2) and loss of normal alveolar structure (grade 3). Lung neutrophil recruitment was evaluated by immunohistochemical staining against myeloperoxidase (MPO) using an anti-MPO antibody (Thermo Scientific). The number of MPO positive cells in three randomly chosen high-power fields was counted and averaged for each animal.

### Bronchoalveolar Lavage

Four animals of each genotype were treated with LPS as described. After 24 hours, mice were anesthetized and a tracheostomy performed. Lungs were lavaged with three aliquots (700 microliters) of sterile saline. Neutrophils in the recovered bronchoalveolar lavage fluid (BALF) were counted in a hemocytometer.

### Flow Cytometry

For quantification of cell populations, additional mice from both genotypes and treatments were studied. After sacrifice, the left lung was washed in sterile PBS immediately after removal, cut in sections and manually homogenized. The resulting extracts were centrifuged, resuspended in 100 microliters of PBS and incubated with fluorescence-labeled anti-CD45, antiCD11b, anti-Gr1 and anti-Ly6G antibodies (BD Biosciences). Cell populations were identified using a FACScanto flow cytometer (BD Biosciences).

### DiGE Analysis

Lungs from WT and KO mice were perfused and then rinsed in TAM (10 mM TRIS-HCl pH 8.5, 5 mM magnesium acetate) and homogenized manually at room temperature in TUCT (7 M urea, 2 M tiourea, 4% CHAPS, 30 mM TRIS- HCl pH 8.5). 50 µg of each sample were covalently labeled with 400 pmol of a specific fluorophore (GE Healthcare): CyDye 3 (WT sample), CyDye 5 (KO sample) and CyDye 2 (pool of WT and KO sample 1∶1). Labeled samples were combined and UCDA (8 M urea, 4% CHAPS, 130 mM DTT, 2% IEF buffer) was added in a 1∶1 ratio. Samples were isoelectrofocused (voltage in gradient for 26 hours at 18°C) in 24 cm pH 3–11 NL strips following manufacturer’s instructions (GE Healthcare). Once the IEF step finished, strips were equilibrated for 15 min in SES (6 M UREA, 30% glycerol, 2% SDS, 75 mM TRIS-HCl pH 6.8), 0.5% DTT and bromophenol blue, and for another 15 minutes in SES+4.5% iodoacetamide and bromophenol blue. Then, they were mounted on top of a 13% SDS–PAGE with stacking gel in a Hoefer S600 apparatus (Ettan DALT Six, GE Healthcare). Electrophoresis was performed at 80 V overnight in the dark at 18°C. After SDS–PAGE, cyanine dye-labeled proteins were visualized directly by scanning using a Typhoon 9400 imager (GE Healthcare). The scanned gels were then directly analyzed with Progenesis SameSpots software (Nonlinear dynamics) and stained with SYPRO Ruby (Molecular Probes).

### Tryptic Digestion and MALDI-ToF Analysis

Differential spots were manually excised over a transilluminator. Gel pieces were washed twice with 180 µL of 25 mM ammonium bicarbonate/acetonitrile (70∶30), dried for 15 min at 90°C, and incubated with 12 ng/µL trypsin (Promega) in 25 mM ammonium bicarbonate. The digestion was allowed to proceed for 1 h at 60°C. Peptides were purified with ZipTip C18 (Millipore) and eluted with 1 µL of CHCA (α-cyano-4-hydroxycinnamic acid) to be placed onto MALDI-ToF́s plate. Once dried, they were analyzed by mass spectrometry on a time-of-flight mass spectrometer equipped with a nitrogen laser source (Voyager-DE STR, Applied Biosystems). Data from 200 laser shots were collected to produce a mass spectrum. Data explorer version 4.0.0.0 (Applied Biosystems) was the software used to analyze the spectra.

### Western Blotting

Tissues were homogenated in a standard RIPA buffer (100 mM TRIS pH 7.4, 150 mM NaCl, 1 mM EDTA, 1% deoxycholic acid, 1% Triton X-100, 0,25% SDS, 1 mM ortovanadate and a protease inhibitor cocktail) and the protein content measured (BCA kit, Pierce, USA). Twelve micrograms of protein or 4 microlitres of serum were loaded in 12% SDS-PAGE or 16.5% Tris-tricine gels and electrophoresed. Then, proteins were transferred to PVDF membranes, blocked in non-fat milk or bovine albumin as needed, and incubated with antibodies against S100A6 (R&D Systems), S100A8 (R&D Systems), S100A9 (R&D Systems), MIP-2 (AbD serotec), LIX (Peprotech), MIP-1α (Abcam), IL-10 (Abcam), p65 (phosphorylated and total, Abcam), p52 (Cell signaling) and actin (Santa Cruz Biotechnology #SC1616). Proteins were then detected by chemoluminiscence (Millipore) using secondary peroxidase-linked antibodies. The resulting images were acquired with a LAS-3000 camera and analyzed using ImageJ software (NIH, USA).

### Quantitative RT-PCR Analysis

RNA was extracted from frozen lung tissue using the Trizol reagent. One microgram of RNA was used to synthesize cDNA using superscript II reverse transcriptase following manufacturer’s instructions. Then, a quantitative PCR was performed using 20 ng of cDNA and TaqMan Universal PCR master mix and the specific TaqMan probes for S100A8 (Mm00496696_g1) and S100A9 (Mm00656925_m1) genes (Applied Biosystems). These probes span along two exons of the gene, thus avoiding the amplification of genomic DNA. Samples were studied in triplicate in an Applied Biosystems 7300 real-time PCR system. Beta-actin was used as control and the relative expression of the analyzed genes was calculated according to manufacturer’s instructions.

### Gelatin Zymography

Activity of matrix metalloproteinases −2 and −9 was measured by standard gelatin zymography as previously described [12]. Briefly, lung homogenates were loaded in a 8% SDS-PAGE gel containing 0.2% gelatin and electrophoresed. The gels were washed in 2.5% Triton X-100 and incubated in a buffer (20 mM TRIS, 5 mM CaCl2, pH 7.4). After staining with Commassie blue and destaining with a methanol/acetic acid mixture, gelatinolytic activity was identified as white bands over a blue background. Gels were scanned and quantified using ImageJ software.

### Statistical Analysis

Data are expressed as mean±SEM. Differences among groups were evaluated using a two-way ANOVA, including genotype and treatment (LPS or saline) as factors. Post-hoc tests were done using the Bonferroni’s correction. A p value lower than 0.05 was considered significant.

## Results

### Increased Lung Inflammatory Response in Mmp8^−/−^ Mice

**Figure 1:**
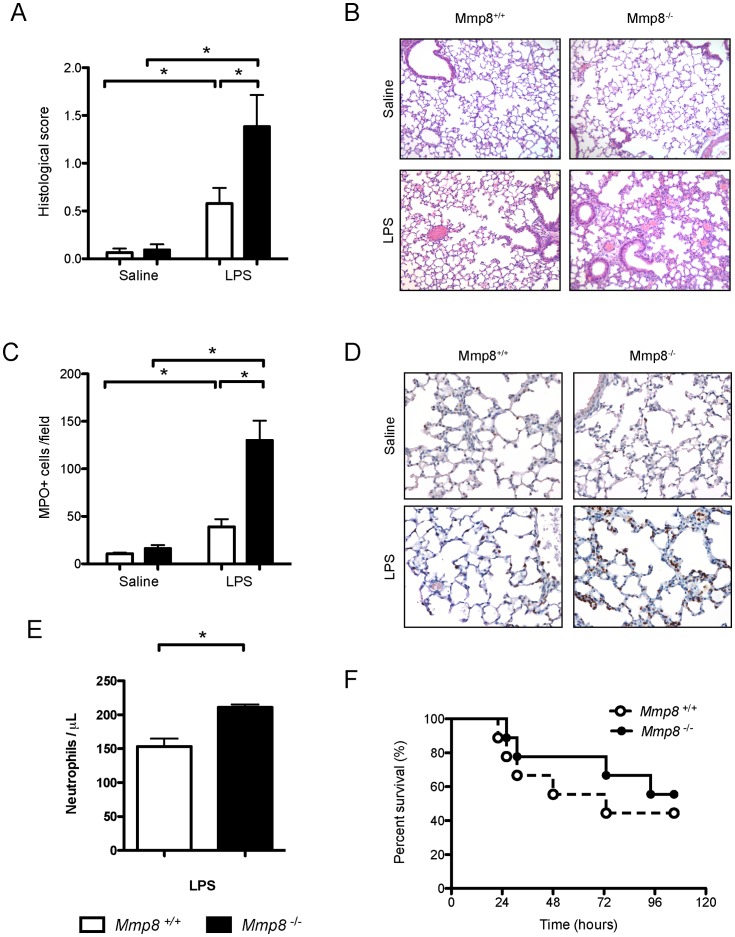
Lung inflammation during endotoxemia. N = 7/group. LPS injection increases histological damage (A-B) and neutrophilic infiltration (C-E). Mice lacking MMP-8 show a more severe injury with increased neutrophils within the lung tissue (C-D) and bronchalveolar lavage fluid (N = 4/group, E). However, there were no differences in survival (F, n = 9 per genotype, log rank test p = 0.52). *p<0.05 in post-hoc tests.

Seven LPS-treated and 7 saline-treated animals per genotype were studied. Treatment with LPS induced histological lung injury in both genotypes (Figure 1A–B), when compared to baseline (p = 0.042 and p = 0.001 for wildtype and knockout mice, respectively). However, the severity of injury in knockout mice doubled that from wildtype counterparts (p = 0.004 for the difference between genotypes in LPS-treated animals). There were no differences between genotypes in saline-treated animals.

Neutrophil infiltration within the lungs was measured by counting MPO-positive cells in immunohistochemical preparations (n = 7/group, Figure 1C–D). As expected, there were no differences in saline-treated animals. LPS administration induced a small but significant increase in lung neutrophils in *Mmp8^+/+^* animals (p<0.05 *vs* saline-treated wildtype mice), and a three-fold higher increase in *Mmp8^−/−^* mice (p<0.001 *vs* saline treated knockout mice, p<0.001 *vs* LPS-treated wildtype mice). There were no differences in leukocyte populations measured by flow cytometry or in the accumulation of myeloid-derived suppressor cells (defined as CD45+, Gr1+, Mac-1+, 28±2% vs 27,2±7% after LPS in wildtype and knockout mice, respectively; n = 5/group, p = 0.92). Neutrophil count in the BALF was also higher in *Mmp8^−/−^* animals than in their wildtype counterparts (Figure 1E). There were no differences in the number of macrophages (data not shown). In spite of the differences in lung injury and inflammation, there were no significant differences in survival between genotypes (Figure 1F).

### Proteomic Analysis of Lung Tissue

**Figure 2:**
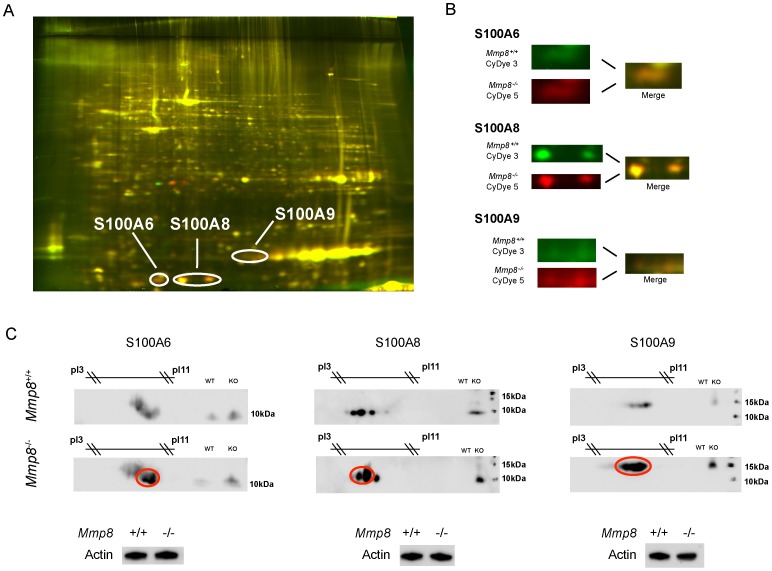
Proteomic identification of S100 proteins in lung from LPS treated animals. A 2D protein electrophoresis (DiGE) was performed with samples from both genotypes (A). Differential spots corresponding to S100A6, S100A8 and S100A9 were identified (B) and validated by western blotting (C). Red circles show the differential spots in 2D western blots.

To identify MMP-8 substrates responsible for the differences in leukocyte infiltration, lung tissue homogenates were analyzed using 2D-DIGE (n = 4/group). Interestingly, members from the S100 protein family, involved in the inflammatory response, were identified, so we focused on these molecules as putative mediators responsible for the differences. Figure 2 shows a representative 2D gel (A) together with the protein spots (B) and confirmatory western blots (C).

### S100 Proteins are Increased in Lungs of MMP-8-deficient Mice

**Figure 3:**
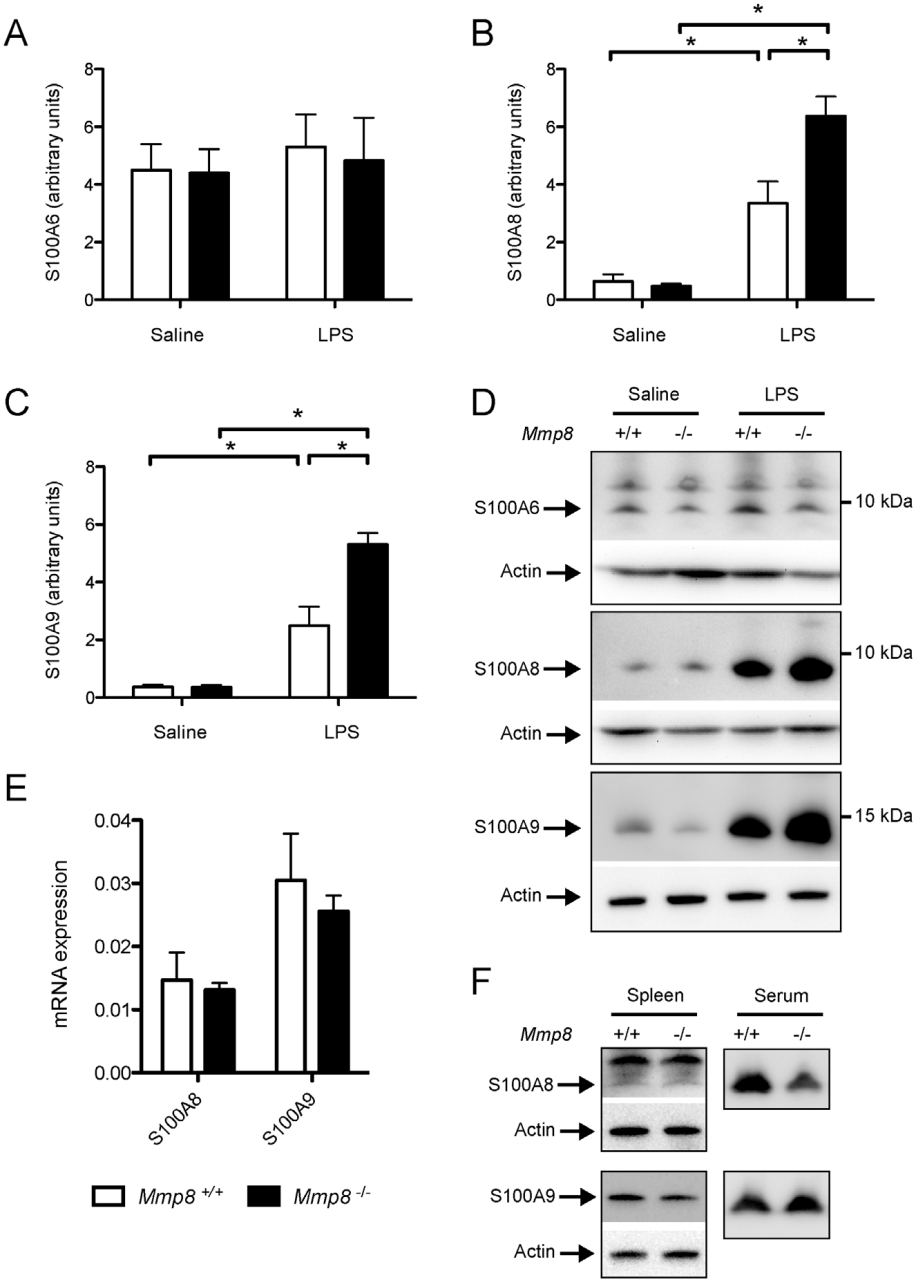
Differential expression of S100 proteins between genotypes. N = 7/group. The differences in S100A6 levels were not confirmed (A, p = 0.45 in the ANOVA). However, S100A8 (B) and S100A9 (C) protein levels increased after LPS injection in both genotypes (p<0.05 in all post-hoc tests). Mice lacking MMP-8 showed significantly higher levels of these two proteins than their wildtype counterparts (p<0.01 and p<0.001 for the differences between genotypes in S100A8 and S100A9 respectively). Panel D shows representative western blots. However, S100A8 and S100A9 gene expression was not different between genotypes during endotoxemia (n = 6/group, E). To discard systemic differences in alarmins, levels of S100A8 and S100A9 were measured in spleen homogenates (n = 7/group) and serum (n = 4/group) from LPS-treated animals, with no significant differences between genotypes (F). *p<0.05 in post-hoc test.

To confirm the results of the proteomic analysis, we performed western blot experiments using lung tissue homogenates (n = 7/group) and antibodies against S100A6, S100A8 and S100A9. There were no differences in S100A6 protein (Figure 3A, p = 0.45 in ANOVA). Regarding S100A8, we did not observe any difference between genotypes in saline-treated animals, but a significant increase after LPS injection (Figure 3B, p<0.001 and p = 0.015 for wildtype and knockout mice respectively). Moreover, S100A8 levels were significantly higher in knockout mice (p = 0.004 for the difference between genotypes). S100A9 showed a similar pattern (Figure 3C). Representative western blots are presented in Figure 3D. S100A8 and S100A9 protein levels were strongly correlated with the leukocyte count in histological sections (correlation coefficients of 0.80 and 0.78 for S100A8 and S100A9 respectively, p<0.01 in both cases).

*S100a8* and *S100a9* gene expression was also studied by quantitative PCR (n = 6/group). Crossing thresholds were 21.779±1,048, 28.125±0.702 and 27.062±0.629 for *actin*, *S100a8* and *S100a9* respectively. The results for each genotype are shown in figure 3E. There were no differences between LPS-treated wildtype and knockout mice (p = 0.531 and p = 0.776 for S100A8 and S100A9 in the ANOVA, respectively).

Finally, we measured S100A8 and S100A9 levels in serum and spleen homogenates to check if the differences observed in lung tissue are a local phenomenon or the manifestation of a systemic difference in alarmin levels. There were no differences in these two molecules in serum or spleen (Figure 3F). These results suggest that the differences observed in protein abundance are not caused by a differential gene expression, but a different protein clearance in lung parenchyma.

### MMP-2 or MMP-9 do not Compensate the Absence of MMP-8

**Figure 4:**
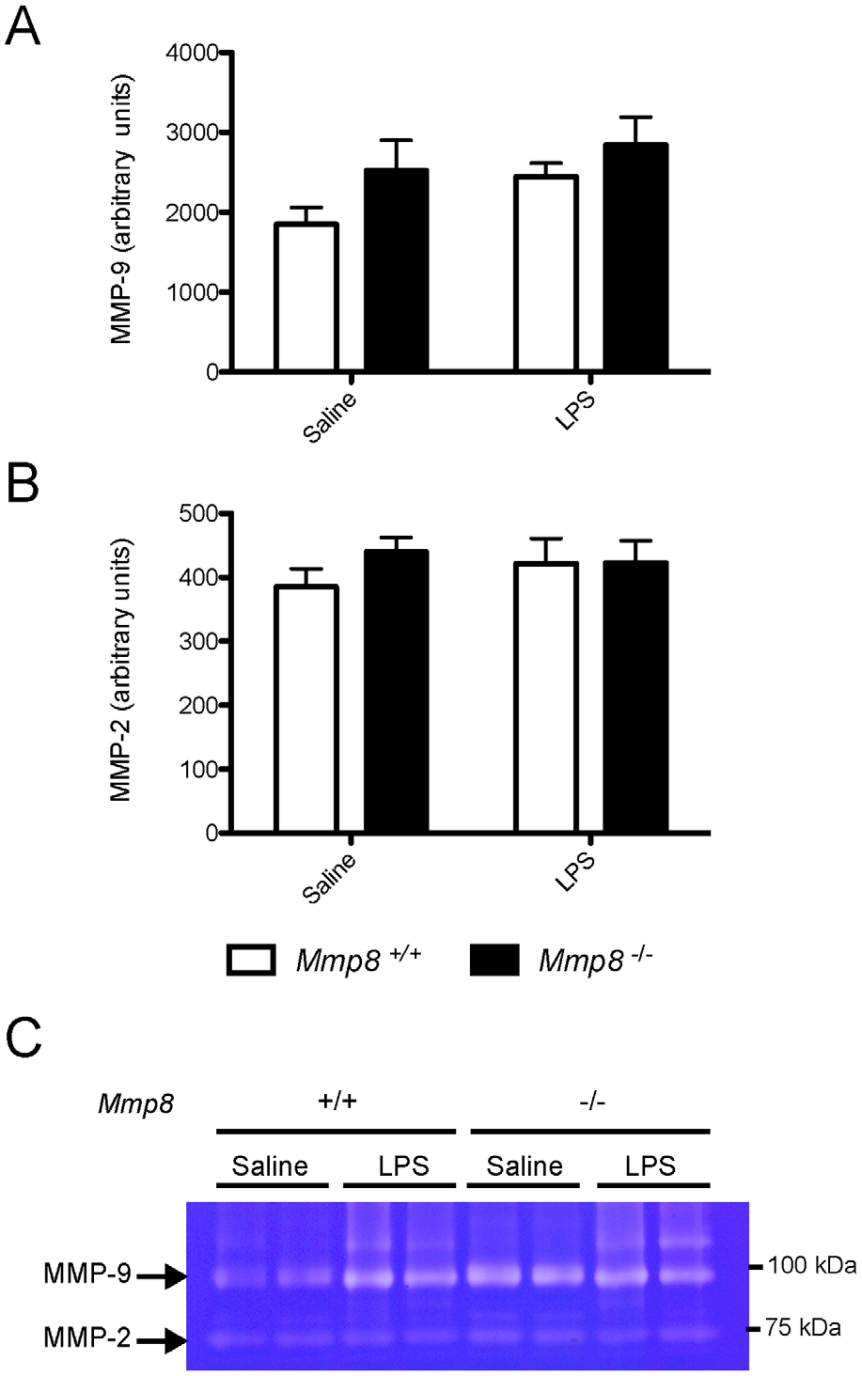
Absence of compensatory changes in MMP-9 (A) or MMP-2 (B) in mice lacking MMP-8 (n = 7/group). A representative zymography is shown in panel C.

As gelatinases MMP-2 and -9 have been involved in processing of S100A8 and S100A9, we studied their levels by gelatin zymography (n = 7/group, Figure 4). There was a non-significant trend to higher levels of MMP-9 in lung tissue after LPS treatment, with no differences between genotypes (p = 0.791 in ANOVA). There were no differences in MMP-2 in response to LPS injection in any of the genotypes. Figure 4C shows a representative zymography.

### Absence of MMP-8 affects Multiple Immune Mediators

**Figure 5:**
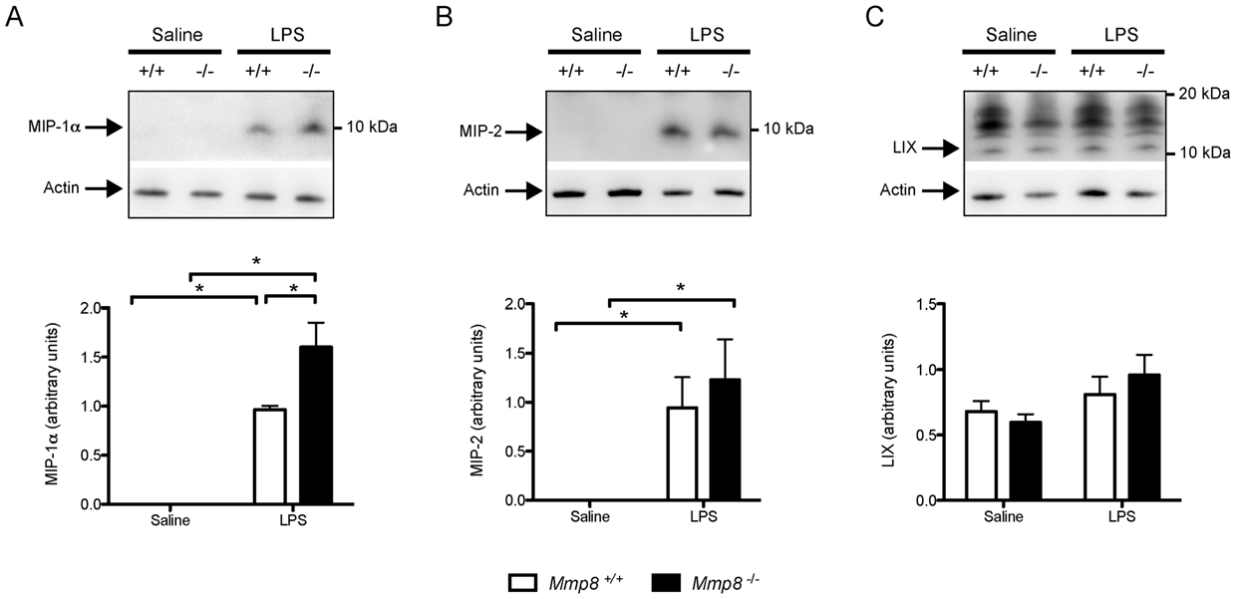
Chemokine levels in lung tissue. N = 7/group. LPS injection increased levels of MIP-1α (A, p<0.001 and p<0.001 for wildtype and knockout mice), and MIP-2 (B, p<0.01 and p = 0.01 for wildtype and knockout mice) but not LIX (C, p = 0.56 in the ANOVA). Moreover, MIP-1α was significantly higher in mice lacking MMP-8 (p<0.01 for the comparison between genotypes). *p<0.05 in post-hoc test.

It has been reported that MMP-8 can process a number of immune mediators, including chemokines responsible for the leukocyte infiltration. To study these factors that were not identified by our proteomic approach, we measured the abundance of MIP-1α, MIP-2 and LIX in lung tissue homogenates (n = 7/group). MIP-1α increased in both genotypes after LPS injection. This increase was more pronounced in mice lacking MMP-8 (Figure 5A). In opposite, there were no differences between genotypes in MIP-2 levels (Figure 5B). Likewise, we did not observe changes in LIX abundance in any experimental group (Figure 5C).

### Activation of Non-canonical NF-κB Pathway in Mmp8^−/−^ Mice

**Figure 6:**
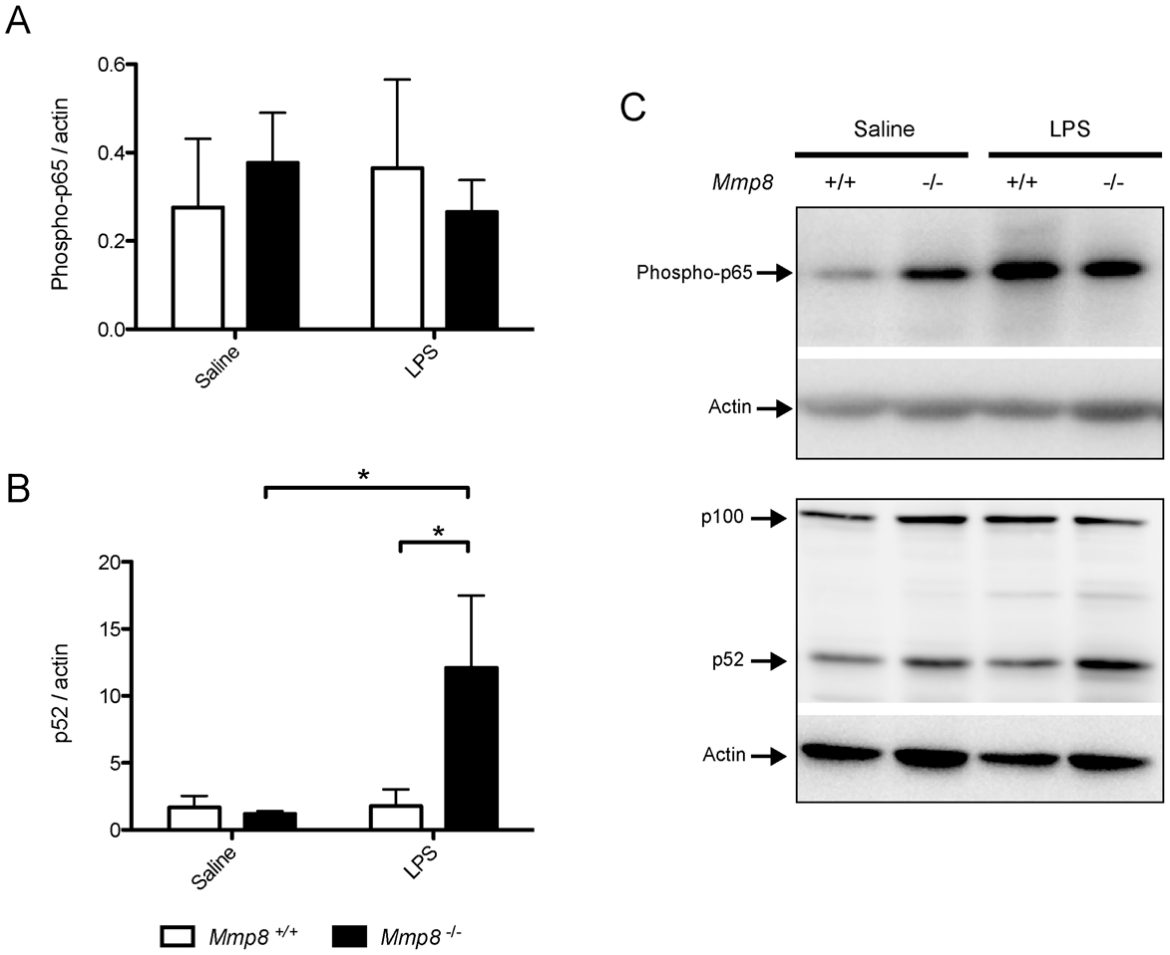
Non-canonical NF-κB activation in LPS-treated, *Mmp8^−/−^* mice. The lung levels of p52 increased significantly in knockout mice after LPS challenge (n = 7/group, p = 0.01 vs saline-treated knockout mice, p<0.02 vs LPS-treated wildtype animals). *p<0.05 in post-hoc test.

The NF-κB pathway is one of the main intracellular triggers of the inflammatory response. Activation of this route was evaluated by western blotting against p65 and p52 (markers of canonical and non-canonical NF-κB pathway respectively). Seven mice per group were studied. There were no differences between genotypes in p65 phosphorylation (Figure 6A). However, there was a marked activation of the non-canonical NF-κB pathway, demonstrated by a 10-fold increase in p52 levels only in mice lacking MMP-8 (Figure 6B).

## Discussion

Our results demonstrate that the absence of MMP-8 increases the neutrophilic lung infiltration after LPS injection. This effect could be explained by the accumulation of S100A8 and S100A9 proteins, in addition to other chemokines. These findings highlight the central role of MMP-8 during the regulation of inflammatory cell recruitment to the lungs by processing a variety of immune mediators.

Matrix metalloproteinases have a wide range of substrates that are responsible for their variety of effects. MMP-8, also known as collagenase-2, has emerged as one of the most important regulators of the inflammatory response [5]. Mutant mice lacking MMP-8 show a characteristic inflammatory response, with an initial delay in cell recruitment, but also with a later persistence of the neutrophilic infiltration [6], [13]–[15]. Therefore, absence of this enzyme ameliorates hyperacute inflammation, but also worsens the response later on [7]. Noteworthy, blood cell counts and the migratory properties of neutrophils in knockout mice are normal [8], so the differences between genotypes rely on the regulation of the inflammatory response.

There are several molecular mechanisms that could be responsible for these opposite effects of MMP-8. First, inflammatory cells must degrade the extracellular matrix fibers in order to migrate, so the collagenolytic activity must be essential for neutrophils to reach the injured site [16]. Additionally, it has been reported that this protease can cleave different chemokines such as LIX [10], [17] or MIP-1α [8]. By this proteolytic inactivation, MMP-8 exerts an anti-inflammatory role. Finally, MMP-8 also regulates neutrophil apoptosis, contributing to the persistence of the infiltrate [18].

The results of the present study show that, in absence of MMP-8, there is an accumulation of S100A8 and S100A9. These myeloid-related proteins, included in the alarmins family, are constitutively expressed in neutrophils, representing the 40–50% of the cytoplasmic content [19], and released at the sites of injury. By binding to RAGE and TLR4, they can trigger a proinflammatory response [20]. Both RAGE and TLR4 are widely expressed in the lung tissue, so this pathway is of major relevance during the alveolar inflammatory response after endotoxemia [21]. Moreover, it has been demonstrated that both S100A8 and S100A9 have chemotactic properties that favor neutrophilic recruitment to the tissues [22]. Although S100A8 and S100A9 can also mediate the recruitment of myeloid-derived suppressor cells [23], with anti-inflammatory properties, we did not find the accumulation of these cells in the lung tissue in our model irrespective of the genotype.

There is increasing evidence that MMPs can regulate the alarmins/RAGE pathway by different mechanisms [24]. MMP-3 and -13 can release RAGE from alveolar epithelial cells [25]. The resulting soluble RAGE could act as a decoy receptor with anti-inflammatory properties. Although MMP-8 shares some structural and functional characteristics with MMP-13 (both are interstitial collagenases), the effects of the former on the release of RAGE have not been addressed. Other MMPs, namely MMP-2 and MMP-9 (gelatinases A and B respectively), can cleave and inactivate S100A8 and S100A9. This mechanism limits the inflammatory response in a model of lung allergic inflammation [26]. The absence of compensatory changes in MMP-2 or MMP-9 supports the role of MMP-8 in the observed differences between genotypes.

In this setting, MMP-8 appears as a central regulator of neutrophilic chemotaxis by its effects on MIP-1α and alarmins. Additionally, MMP-8 can modulate LIX activity and IL-10 levels, as shown in other experimental models [6], [9], [10]. All these signals may result in the activation of the NF-κB route. The chemotactic activity of another RAGE and TLR ligand, HMGB1, has been related to the activation of the non-canonical NF-κB pathway [27]. Our results showing increased levels of p52 in mice lacking MMP-8 resemble this finding. However, we cannot discard that other factors not identified in our study are the cause of the increase in NF-κB activity, as this is a final common pathway in the inflammatory response.

The multiple and opposite effects of MMPs, and MMP-8 in particular, can explain some contradictory results in the literature. Absence of MMP-8 has been related to pro- and anti-inflammatory responses. Regarding to MMP-8 and sepsis, all the experimental models using LPS, either intratracheal [8], [17] or intraperitoneal (present study), report an increase in lung inflammation in knockout mice. Recently, Solan et al. [28] have shown the opposite effect (decreased neutrophilic infiltration and better outcome in Mmp8^−/−^ mice) in a model of cecal ligation and puncture. The differences in the type and severity of injury (with a survival rate in wildtype mice of 40% in our study, but 0% in the peritonitis model), or the involvement of other mediators such as IL-10 (which is increased in knockout mice [9]) may explain these discrepancies. In human sepsis, MMP-8 correlates with severity, mortality and organ failures [28], [29]. Therefore, targeting this enzyme could be an interesting therapeutic approach. However, a deeper knowledge of the pro- and anti-inflammatory effects of the enzyme is needed to make any firm recommendation.

In conclusion, the results described here reinforce the central role of MMP-8 in the regulation of neutrophil recruitment to the lungs during sepsis, adding the myeloid-related proteins S100A8 and S100A9 as one of the involved mediators. This anti-inflammatory role of MMP-8 must be considered before proposing anti-MMP strategies in sepsis.
